# Mediation of Resilience in the Association Between Personality Traits and Suicidal Ideation Among Chinese Adolescents

**DOI:** 10.3389/fpsyg.2022.898318

**Published:** 2022-06-03

**Authors:** Yusan Che, Die Fang, Le Cai, Hailiang Ran, Lin Chen, Sifan Wang, Junwei Peng, Hao Sun, Xuemeng Liang, Yuanyuan Xiao

**Affiliations:** Department of Epidemiology and Health Statistics, School of Public Health, Kunming Medical University, Kunming, China

**Keywords:** personality traits, suicidal ideation (SI), resilience, adolescents, mediation

## Abstract

A positive connection has been established between personality traits and suicidal ideation (SI). However, the possible mediation of resilience within this association has never been thoroughly discussed. In this study, we aimed to investigate this topic by using population-based cross-sectional survey data of 4,489 Chinese children and adolescents. A self-administered questionnaire was used to collect information from the participants. Univariate and multivariate logistic regression models were adopted to measure the crude and adjusted associations between personality traits, SI, and resilience. Path analysis was performed to evaluate the mediation of resilience in the association between personality traits and SI. For 4,489 study subjects, the mean age was 13.4 years, ethnic minorities accounted for 71.8%, and over a half (54.6%) were middle school students. The reported prevalence rates for 1-week, 1-year, and lifetime SI were 27.6% (95% CI: 24.9%, 30.0%), 35.5% (95% CI: 30.8%, 41.0%), and 56.7% (95% CI: 52.3%, 61.0%), respectively. Girls reported a significantly higher prevalence of SI than boys. Path analysis results revealed a prominent mediation of resilience; moreover, for different dimensions of personality traits, the proportion of mediation by resilience varied. Our major findings suggest that resilience-based intervention measures could be considered in preventing personality traits related to suicidal risk among youngsters. For children and adolescents, the measuring of personality dimensions may also be helpful in targeting key subpopulations of intervention priority.

## Introduction

Suicide is a major global health problem, killing about 800,000 people worldwide each year (Naghavi, 2019). A meta-analysis revealed a pooled suicide rate of 3.77/100,000 for adolescents globally, with considerable discrepancies between countries, age groups, and sexes (Glenn et al., 2020). Despite prevention and intervention efforts, the adolescent suicide rate has kept rising in the past decade (Glenn et al., 2015). Moreover, starting from the beginning of 2020, the COVID-19 pandemic further aggravated the situation, as a significant increase in suicide-related behaviors among teenagers had been reported (Hill et al., 2021). Adolescent suicide is by no means a simple public health issue, but also of great social importance. Therefore, it is imperative to prevent suicide among teenagers.

It has been widely accepted that suicide proceeds along a continuum path, starts from suicidal ideation (SI), suicidal plan, and suicidal attempt and ends at suicidal death (Silverman et al., 2007). SI refers to an individual’s thought or cognitive desire to end life (Bonner and Rich, 2011). In the general population, prior SI is one of the strongest predictors of future suicidal attempt and suicidal death (Law et al., 2018; Mars et al., 2019). It is believed that SI is the inevitable psychological stage for individuals who eventually adopt suicidal behavior (Pan et al., 2021). Under this circumstance, intervention on SI can be an effective primary prevention measure for suicide.

Personality traits reflect people’s characteristic patterns of thoughts, feelings, and behaviors. Among various theoretical models for suicide, personality is an identified independent risk factor (Stefa-Missagli et al., 2020). For instance, it has been suggested that people with SI usually showed more introversion, neuroticism, and psychosis (Brezo et al., 2006). Despite the positive connection between personality traits and SI, the inherent nature of personality traits prevents acquired change. However, it would be meaningful to investigate whether other factors, especially modifiable factors, have participated in this association, to provide intervention thoughts in preventing personality traits associated with suicidal risk.

Resilience is a hot topic in the field of positive psychology (Bolier et al., 2013). It refers to an individual’s ability to successfully handle or cope with adverse events (Rutter, 1987). In their previous study, Chang et al. (2021) found a negative association between resilience and SI among adolescents. Also, it had been reported that along with the increase in resilience level, the SI risk was reduced (Kim, 2021). Moreover, the relationship between resilience and personality traits had also been discussed. Two previously published studies revealed that, among all dimensions of personality traits, psychoticism, introversion, neuroticism, and conscientiousness were significantly related to resilience (Campbell-Sills et al., 2006; Annalakshmi, 2007). These positive findings remind us that resilience may play a mediation role in the association between personality traits and SI. However, this reasonable assumption has never been thoroughly discussed in adolescents.

Considering the novelty and significance of this proposed hypothesis, and the fact that individual resilience can be considerably enhanced through psychological or psychosocial measures (Campbell-Sills et al., 2006), in this study, we aimed to preliminarily explore the possible mediation of resilience in the association between personality traits and SI in a large representative sample of Chinese adolescents.

## Materials and Methods

### Study Design

After the approval of the Ethics Review Board of Kunming Medical University, we conducted a cross-sectional survey in Kaiyuan city, Honghe Hani, and Yi Autonomous Prefecture, Yunnan Province, China. Unlike other Han majority-inhabited regions in Yunnan, based on the official population statistics, the proportion of ethnical minorities in Kaiyuan surpassed 60% (Xinhuanet, 2018). The survey was from 19 October to 3 November 2020. The sample size was preliminarily calculated by using the formula for a simple random sampling method. The prevalence of SI varied for a variety of instruments had been used, based on findings of domestic studies, and we set a conservative estimated SI prevalence of 12% (Wan et al., 2016), with an acceptable error of 1.5% and an effective response rate of 90%, and got a preliminarily calculated sample size of 2005. Since the sampling error of multistage cluster sampling is bigger than that of simple random sampling, we used a conservative design effect value of 2 for further adjustment; therefore, the final calculated sample size was 4010.

A two-stage probability proportionate to sample size (PPS) sampling method was used to determine survey participants. Prior to the sampling process, by using the predefined inclusion and exclusion criteria, we asked all schools in Kaiyuan to report detailed information on the exact count of eligible students for every class. Based on this information, we successfully calculated the required proportions for sampling. For the implementation of sampling, at first, based on the calculated proportion, 19 schools within Kaiyuan were randomly selected, which include 8 primary schools, 9 middle schools, and 2 high schools. After that, within each chosen school, by using the proportions of grades, within each grade, two or three classes were randomly selected, and all students within the chosen class who satisfied the inclusion criteria were eligible participants. The inclusion criteria for participants are as follows: (1) above 10 and below 18 years old and (2) signed consents from the legal guardians. Participants were excluded if any of the following conditions were satisfied: (1) cognitive disorder; (2) severe physical illness; and (3) refusal to participate.

### Variables and Definitions

A comprehensive questionnaire was used to collect relevant information from the participants, and it mainly includes the following parts: general characteristics (demographics, familial features, and socioeconomic status), SI, resilience, personality traits, depression and anxiety symptoms, and self-harm behaviors. Except for the self-developed general characteristics part, which asks factual questions, the rest of the information was measured by using established instruments of ideal reliability and validity.

#### Personality Traits

We used Eysenck Personality Questionnaires (EPQ; Gong, 1983). It has 88 questions with each scoring 1 or 0. The whole instrument can be further divided into four dimensions (i.e., EPQ-P, EPQ-E, EPQ-N, and EPQ-L): EPQ-P contains 23 items, mainly used to measure underlying mental traits, and it is also known as stubbornness. EPQ-E has 21 items, measuring introversion or extroversion. EPQ-N includes 24 items, measuring an individual’s neuroticism-emotional stability, with a higher combined score indicating a stronger tendency to neuropathy. EPQ-L is a validity scale that contains 20 items, reflecting the truthfulness of the answers: if a person gets an L score over 61.5, then this person should be deleted from the final analysis. Meanwhile, it also represents a stable personality function. EPQ-L is used to test the truth or falsity of the response, and therefore, its information was not included in the analysis.

A person’s temperament is jointly determined by EPQ-E and EPQ-N (Cao et al., 2009): if E, N scores are from 43.3 to 56.7, this person is labeled as the middle type; if E, N scores are from 38.5 to 43.3 or from 56.7 to 61.5, the respondent will be labeled as tendentiousness; if the two scores are below 38.5 or above 61.5, the individual is categorized as the typical type. Based on this criterion, we grouped our participants into five temperament types: choleric (extrovert and emotionally unstable), sanguineous (extrovert and emotional stable), phlegmatic (introvert and emotionally stable), melancholic (introvert and emotionally unstable), and middle type (Chang et al., 2012). Previous studies have used Eysenck Scale in children and adolescents (Chen et al., 2016). The Cronbach’s α of EPQ for the current analytical sample is 0.89 (Bootstrap 95% CI: 0.88, 0.90).

#### Resilience

Resilience was measured by using the Resilience Scale for Chinese Adolescents (RSCA) designed by Hu and Gan (2008). This scale includes 27 questions, and any question has five options rated from 1 to 5. A higher combined score of RSCA suggests better resilience. There are five dimensions within RSCA: goal concentration, emotion regulation, family support, positive perception, and interpersonal assistance. The Cronbach’s α of RSCA for the current analytical sample is 0.85 (Bootstrap 95% CI: 0.84, 0.86).

#### Suicidal Ideation

We used the Beck Scale for Suicide Ideation (BSSI) to gauge 1-week and lifetime SI (Beck et al., 1988). Participants who answered “weak, no desire to live/the wish to die is strong/more would be dead than alive/the desire to attempt suicide is strong/a strong wish to be killed by external force” in the previous week or ever were deemed 1-week suicidal ideators or lifetime suicidal ideators. The Cronbach’s α of BSSI for the current analytical sample is 0.86 (Bootstrap 95% CI: 0.87, 0.88). The 1-year SI was determined by using the Suicidal Behaviors Questionnaire-Revised (SBQ-R; Osman et al., 2001), for a specific question on 1-year SI inside SBQ-R; participants answered “never happens” were negative, and otherwise were positive. The Cronbach’s α of SBQ-R for the current analytical sample is 0.87 (Bootstrap 95% CI: 0.86, 0.88).

#### Depression and Anxiety Symptoms

The depression scale (PHQ-9) consists of nine items, and each item has four options, with scores assigned from 0 to 3. PHQ-9 has good reliability and validity in the assessment of adolescent depression mood, and the recommended cut-off for PHQ-9 is 5 (Spitzer et al., 1999). The Cronbach’s α of PHQ-9 for the current analytical sample is 0.88 (Bootstrap 95% CI: 0.86, 0.89). The Anxiety Scale (GAD-7) consists of seven questions with 4-point Likert style responses (Spitzer and Kroenke, 2006). The total score of GAD-7 is from 0 to 21, and a cut-off of 5 is the most commonly used. The Cronbach’s α of GAD-7 for the current analytical sample is 0.91 (Bootstrap 95% CI: 0.90, 0.92).

#### Self-Harm Behaviors

A revised version of the Adolescent Self-Injury Scale (MASH) developed by Feng (2008) was used to measure the lifetime frequency and severity of the 18 most commonly seen SH behaviors among Chinese adolescents. The frequency of SH is categorized as never, once, two to four times, five times, and above. The severity of SH is defined as non-observable, slight, medium, severe, and critical.

### Statistical Analysis

Descriptive analysis was used to describe the general characteristics of the participants. Crude and adjusted associations between personality traits, resilience, and three types of SI (i.e., 1-week SI, 1-year SI, and lifetime SI) were explored by using univariate and multivariate logistic regression models. Path analysis was performed to explore the mediation of resilience in the association between personality traits and all three types of SI. Personality traits were divided into different temperament types (choleric, sanguineous, phlegmatic, melancholic, and middle type) in univariate and multivariate models. In the path model, personality traits entered the model in the form of quantitative data (i.e., EPQ-P, EPQ-E, and EPQ-N). All statistical analyses were executed by using the R software (Version 4.0.2). Due to the internal correlation caused by clustering sampling, survey data-related packages were used throughout. Except for univariate logistic regression models, which adopted a comparatively lower significance level of 0.10 to screen for potential covariates, for all the rest statistical analyses, a two-tailed *p*-value less than 0.05 was deemed statistically significant.

## Results

### General Characteristics of Study Participants

In this survey, we initially identified a total of 4,943 participants, but 85 students did not return signed informed consent and refused to participate, with an informed consent return rate of 98.28%. Among participants who returned informed consents signed by parents or guardians, during the data cleaning stage, 94 were found to be younger than 10 or older than 18. In addition, 260 respondents were deemed untruthful in providing the answers, since their EPQ-L scores were over 61.5, and 6 respondents had missing information in critical variables. After the exclusion of all ineligible respondents, we included the rest 4,489 subjects in the final analysis, with an effective response rate of 90.82%. The basic characteristics of the 4,489 respondents are shown in Table 1: boys and girls were about equivalent in numbers (2,208 versus 2,281); the mean age for all participants was 13.43 years, with a standard error (SE) of 0.4. As to the proportions of ethnicity, Han majority accounted for 28.2%, and the top three minorities were Yi (39.8%), Miao (14.7%), and Hui (5.2%). Most subjects were middle school students (54.6%), and the age means for their fathers and mothers were 41.5 (SE: 0.1) and 39.1 (SE: 0.2) years, respectively.

**TABLE 1:** General characteristics of 4,489 study subjects.

Characteristics	*N* (%)	Mean (SE)^§^ /Median (IQR)^¶^
**Sex**		
Boys	2208 (49.2)	
Girls	2281 (50.8)	
Age		13.4 (0.4)^§^
**Ethnicity**		
Han majority	1266 (28.2)	
Yi minority	1785 (39.8)	
Miao minority	660 (14.7)	
Hui minority	236 (5.2)	
Other minorities	542 (12.1)	
**Grade**		
Primary school	1530 (34.1)	
Middle school	2450 (54.6)	
High school	509 (11.3)	
**Single child of the family**		
Yes	985 (21.9)	
No	3504 (78.1)	
**Father’s education level**		
Illiteracy	198 (4.4)	
Primary school	1386 (30.9)	
Middle or high school	1883 (41.9)	
College or above	287 (6.4)	
Unknown or missing	735 (16.4)	
Father’s age		41.5 (0.1)^§^
**Mother’s education level**		
Illiteracy	546 (12.2)	
Primary school	1285 (28.6)	
Middle or high school	1626 (36.2)	
College or above	225 (5.0)	
Unknown or missing	807 (18.0)	
Mother’s age		39.1 (0.2)^§^
**Marital status of the parents**		
In marriage	3796 (84.6)	
Divorced or widowed	477 (10.6)	
Remarried with someone else	216 (4.8)	
Depression: Yes (PHQ-9 ≥ 5)	1572 (35.0)	
Anxiety: Yes (GAD-7 ≥ 5)	1096 (24.4)	
Self-harm: Yes	1782 (39.7)	
**Personality traits**		
EPQ total score		196.7 (28.5)**^¶^**
EPQ-P score (Psychoticism)		48.5 (11.2)**^¶^**
EPQ-E score (Extraversion)		53.6 (16.6)**^¶^**
EPQ-N score (Neuroticism)		41.7 (21.1)**^¶^**
Temperament types		
**Middle type**	1422 (31.7)	
Choleric temperament	720 (16.0)	
Sanguineous temperament	1232 (27.4)	
Phlegmatic temperament	620 (13.8)	
Melancholic temperament	495 (11.1)	
**Resilience**		
RSCA total score		89 (19)**^¶^**
Goal concentration		17 (6)**^¶^**
Emotion regulation		20 (8)**^¶^**
Family support		21 (6)**^¶^**
Positive perception		14 (5)**^¶^**
Interpersonal assistance		20 (6)**^¶^**

*^§^mean (SE) and ^¶^median (IQR).*

The medians for P, E, and N dimensions of personality were 48.5 (IQR: 11.2), 53.6 (IQR: 16.6), and 41.7 (IQR: 21.1), respectively. Based on E and N scores, all subjects were divided into five different temperament types, and the middle type (*N* = 1,422) accounted for the largest proportion (31.7%). The median resilience for all participants was 89 (IQR: 19). The reported prevalence rates for 1-week, 1-year, and lifetime SI were 27.6% (95% CI: 24.9%, 30.0%), 35.5% (95% CI: 30.8%, 41.0%), and 56.7% (95% CI: 52.3%, 61.0%), respectively. Girls reported significantly higher (*p* < 0.05) prevalence of all three types of SI than boys: 31.3 to 23.7% for 1-week SI, 43.4 to 27.4% for 1-year SI, 63.2 to 50.0% for lifetime SI.

### Personality Traits, Resilience, and Suicidal Ideation

The crude and adjusted associations between personality traits and different types of SI were estimated by using univariate and multivariate logistic regression models, and the results were collectively displayed in Table 2. For 1-week SI, univariate analysis showed that sex, depression, anxiety, temperament types, SH, and resilience were among the identified covariates. After further adjusted by using the multivariate model, personality traits (measured by temperament types) were significantly associated with 1-week SI: compared with the middle type, sanguineous temperament was associated with reduced risk of SI (OR: 0.59, 95% CI: 0.44, 0.79); phlegmatic (OR: 1.30, 95% CI: 1.02, 1.67) and melancholic temperament (OR: 1.67, 95% CI: 1.34, 2.09) were associated with increased risk of SI. Resilience was inversely associated with 1-week SI: compared with subjects of lower resilience (defined as RSCA < median), subjects of higher resilience (defined as RSCA ≥ median) were observed with a decreased risk of 1-week SI (OR: 0.47, 95% CI: 0.39, 0.57).

**TABLE 2:** Univariate and multivariate logistic regression models fitting results for associated factors of different types of SI.

Covariates	1-week SI		1-year SI		Lifetime SI	
	Univariate	Multivariate	Univariate	Multivariate	Univariate	Multivariate
	OR (90% CI)	OR (95% CI)	OR (90% CI)	OR (95% CI)	OR (90% CI)	OR (95% CI)
Sex (Ref: Boys): Girls	1.46 (1.31, 1.67)	1.38 (1.13, 1.68)	2.04 (1.74, 2.39)	1.76 (1.49, 2.08)	1.72 (1.52, 1.94)	1.64 (1.45, 1.85)
**Ethnicity (Ref: Han majority)**						
Yi minority	1.03 (0.84, 1.26)		0.75 (0.60, 0.92)	0.92 (0.69, 1.22)	0.90 (0.75, 1.09)	
Hui minority	1.06 (0.77, 1.46)		1.18 (0.83, 1.67)	1.22 (0.74, 2.01)	1.09 (0.78, 1.51)	
Miao minority	1.08 (0.87, 1.35)		0.83 (0.62, 1.11)	0.91 (0.64, 1.29)	1.00 (0.74, 1.33)	
Other minorities	1.08 (0.89, 1.32)		0.92 (0.79, 1.07)	1.00 (0.70, 1.42)	0.98 (0.89, 1.09)	
Age: +1 year	0.97 (0.92, 1.03)		1.12 (1.05, 1.19)	0.98 (0.88, 1.09)	1.11 (1.08, 1.15)	0.96 (0.89, 1.03)
**Grade (Ref: Primary school)**						
Middle school	1.07 (0.91, 1.27)		1.60 (1.19, 2.14)	1.15 (0.75, 1.76)	1.57 (1.29, 1.90)	1.24 (0.89, 1.71)
High school	0.79 (0.53, 1.17)		2.09 (1.54, 2.83)	1.16 (0.63, 2.11)	1.88 (1.62, 2.17)	1.41 (0.87, 2.27)
Single child (Ref: Yes): No	1.16 (1.03, 1.31)	1.14 (0.98, 1.32)	0.80 (0.68, 0.92)	0.95 (0.80, 1.13)	1.00 (0.85, 1.17)	
**Father’s education level (Ref: Illiteracy)**						
Primary school	0.87 (0.71, 1.07)		0.83 (0.63, 1.08)	0.82 (0.40, 1.68)	0.96 (0.80, 1.16)	1.00 (0.67, 1.51)
Middle or high school	0.90 (0.71, 1.14)		1.04 (0.80, 1.36)	0.95 (0.48, 1.87)	0.96 (0.76, 1.22)	1.15 (0.70, 1.88)
College or above	0.79 (0.59, 1.05)		1.40 (1.06, 1.85)	1.19 (0.50, 2.80)	1.18 (0.92, 1.52)	1.55 (0.97, 2.48)
Father’s age: +1 year	1.03 (0.95, 1.12)		1.09 (1.02, 1.17)	1.02 (0.98, 1.05)	1.07 (1.02, 1.12)	1.06 (0.97, 1.15)
**Mother’s education level (Ref: Illiteracy)**						
Primary school	1.01 (0.82, 1.25)	1.09 (0.84, 1.41)	0.79 (0.65, 0.95)	0.94 (0.63, 1.38)	0.84 (0.70, 1.00)	
Middle or high school	0.82 (0.69, 0.97)	0.89 (0.67, 1.19)	1.06 (0.86, 1.31)	1.22 (0.75, 2.00)	0.90 (0.75, 1.08)	
College or above	0.85 (0.69, 1.06)	0.88 (0.69, 1.14)	1.42 (1.07, 1.88)	1.19 (0.64, 2.22)	0.94 (0.67, 1.32)	
Mother’s age: +1 year	1.04 (0.97, 1.12)		1.12 (1.04, 1.20)	1.01 (0.97, 1.05)	1.08 (1.00, 1.15)	
**Marital status of the parents (Ref: In marriage)**						
Divorced or widowed	1.46 (1.23, 1.73)	1.47 (1.09, 1.99)	1.49 (1.20, 1.87)	1.34 (0.93, 1.94)	1.12 (0.96, 1.32)	1.04 (0.73, 1.48)
Remarried with someone else	1.40 (1.07, 1.82)	1.20 (0.84, 1.69)	2.04 (1.50, 2.78)	2.25 (1.51, 3.37)	1.33 (1.09, 1.63)	0.92 (0.68, 1.23)
Depression (Ref: PHQ-9 < 5): PHQ-9 ≥ 5	2.93 (2.56, 3.36)	1.03 (0.81, 1.29)	6.44 (5.72, 7.24)	2.06 (1.37, 3.11)	4.59 (3.90, 5.40)	1.83 (1.37, 2.44)
Anxiety (Ref: GAD-7): GAD-7 ≥ 5	4.15 (3.63, 4.73)	2.10 (1.67, 2.63)	6.85 (5.73, 8.18)	1.84 (1.08, 3.15)	5.30 (4.69, 5.99)	1.51 (1.09, 2.09)
Self-harm (Ref: No): Yes	3.05 (2.67, 3.47)	1.88 (1.55, 2.27)	4.58 (3.74, 5.60)	2.94 (2.59, 3.34)	3.75 (3.13, 4.48)	2.49 (1.92, 3.22)
**Temperament types (Ref: Middle type)**						
Choleric	2.16 (1.79, 2.60)	1.03 (0.81, 1.31)	5.68 (4.82, 6.70)	2.58 (2.04, 3.27)	3.28 (2.69, 4.00)	1.46 (1.08, 1.97)
Sanguineous	0.46 (0.38, 0.56)	0.59 (0.44, 0.79)	0.86 (0.72, 1.06)	0.98 (0.77, 1.26)	0.71 (0.61, 0.83)	0.83 (0.61, 1.14)
Phlegmatic	1.55 (1.31, 1.83)	1.30 (1.02, 1.67)	0.96 (0.79, 1.16)	0.91 (0.61, 1.35)	1.14 (1.02, 1.28)	1.25 (1.02, 1.55)
Melancholic	4.47 (3.77, 5.30)	1.67 (1.34, 2.09)	9.58 (7.40, 12.40)	2.84 (2.04, 3.90)	10.93 (7.67, 15.61)	3.48 (1.94, 6.25)
Resilience (Ref: <89): ≥89	0.25 (0.22, 0.29)	0.47 (0.39, 0.57)	0.35 (0.29, 0.42)	0.58 (0.47, 0.72)	0.35 (0.31, 0.41)	0.64 (0.50, 0.80)

For both 1-year and lifetime SI, sex, depression, anxiety, SH, temperament types, and resilience were among the prominent covariates by the univariate model. After further adjusted by using the multivariate model, personality trait was a significant associated factor: compared with the middle type, choleric temperament and melancholic temperament were observed with an increased risk of SI: for choleric temperament, ORs for 1-year SI and lifetime SI were 2.58 (95% CI: 2.04, 3.27) and 1.46 (95% CI: 1.08, 1.97); for melancholic temperament, ORs were 2.84 (95% CI: 2.04, 3.90) and 3.48 (95% CI: 1.94, 6.25). The same inverse associations also existed between resilience and 1-year, lifetime SI: adolescents of higher resilience level found an OR of 0.58 (95% CI: 0.47, 0.72) in 1-year SI and an OR of 0.64 (95% CI: 0.50, 0.80) in lifetime SI.

### Mediation of Resilience in Personality-Suicidal Ideation Associations

**FIGURE 1:**
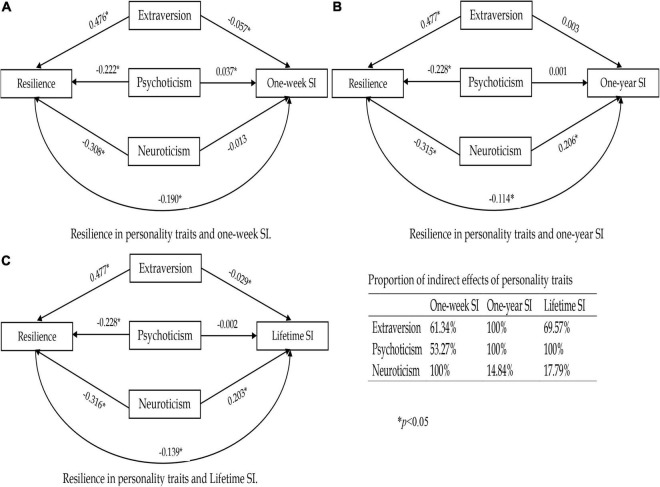
Path models indicating the mediation of resilience in the associations between dimensions of personality traits and SI. **(A–C)** Represent 1-week SI, 1-year SI, and lifetime SI, respectively.

We further analyzed the adjusted association between personality traits and resilience: personality traits were significantly associated with resilience (Supplementary Table 1). All these analytical results suggest a possible mediation of resilience in the association between personality traits and all three types of SI. We further constructed three hypothetical path models to evaluate the possible mediation of resilience, and the results were jointly shown in Figure 1: resilience presented as a prominent mediator in the association between personality traits and all three types of SI mediated 53.27, 100, and 61.34% of the total associations between psychoticism, neuroticism, extraversion, and 1-week SI (the main variables that had been controlled for were sex, whether single child, mother’s education level, and depression); for 1-year SI, after controlled for sex, age, grade, father’s education level, and depression, the mediation proportions were 100, 14.84, and 100%; for lifetime SI, after controlled for sex, grade, age, and depression, the mediation proportions were 100, 17.79, and 69.57%.

**FIGURE 2:**
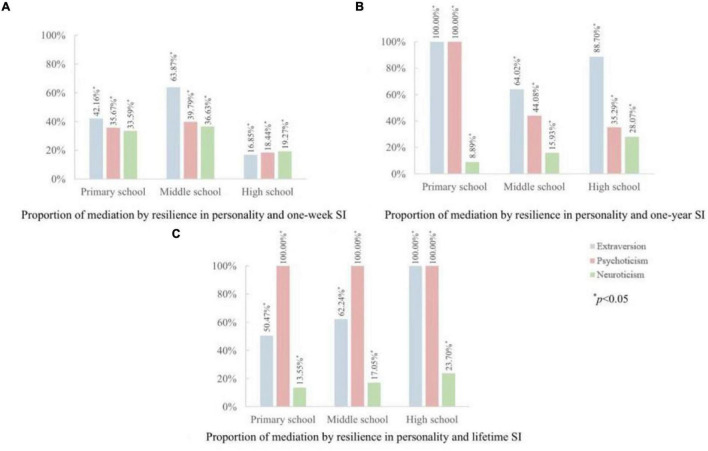
Proportions of mediation by resilience in the associations between personality traits and SI for different types of students. **(A)** One-week SI; **(B)** One-year SI; **(C)** Lifetime SI.

Considering that for primary school, middle school, and high school students, the mediation of resilience in the association between personality traits and SI indicators may be different. We further performed a series of path analyses for the three types of students separately, and the results were jointly displayed in Figure 2. As expected, noticeable disparities were found in mediation by resilience for different types of students surveyed: for 1-week SI, the mediation proportions were generally higher for primary school and middle school students; for 1-year SI, mediation proportions varied for different dimensions of personality traits; for lifetime SI, high school students were observed higher mediation proportions in extraversion and neuroticism dimensions.

## Discussion

In this population-based cross-sectional study of 4,489 Chinese children and adolescents, we found that the prevalence of SI was high, and depression, anxiety, SH, and resilience were among the significantly associated factors of SI. Although in univariate analysis, the grade was significantly associated with SI, after multivariate adjustment, for all three types of SI, the grade presented an insignificant association. More importantly, we found a significant association between personality traits and SI. After classifying personality traits into different temperament types, diverse associations were found for different temperament types and SI. Further analysis revealed a significant mediating role of resilience in the relationship between personality traits and SI. Our major findings have significant implications in guiding resilience-based intervention measures for reducing personality-associated suicidal risk.

After controlling for possible influencing factors, temperament types were significantly associated with SI. For instance, adolescents with melancholic temperament had a higher risk of SI. It has been found that melancholic individuals usually show a pessimistic tendency, and they are more likely to report depression (Stanghellini, 2008). As depression is one of the most prominent risk factors for SI (Roberts and Chen, 1995; Bertule et al., 2021), it is not a surprise for us to find this positive association. In contrast, sanguineous temperament was associated with decreased 1-week SI. Generally, individuals with sanguineous temperament tend to be more extroverted and emotionally stable, and these characteristics are indicative of better mental health (Chen and Chen, 2012) and an identified protective factor of SI (Blüml et al., 2013).

In accordance with our anticipation, path analysis results suggested that resilience could be a significant mediator in the association between personality traits and SI. Evidence from the existing literature can be used to support this mediation of resilience: at first, resilience was related to reduced risk of SI (Pietrzak et al., 2011; Cheung et al., 2019); besides, different personality traits had disparate effects on resilience, for instance, extraversion can increase resilience, while neuroticism will decrease it (Deng et al., 2020). This major finding suggests that personality traits associated with SI can be effectively intervened by promoting resilience. For children and adolescents, resilience can be effectively improved through short-term interventions (Lutha and Cicchetti, 2000). Adolescent resilience can also be improved by creating a good family environment and a positive campus culture, and by improving the social support system (Chang and Pan, 2013). Counseling agencies can also be set up to help young people cope with negative life events to enhance their resilience (Hao and Lei, 2007).

Another important finding of our study is that, for different dimensions of personality traits, resilience played discordant mediation roles: for extraversion and psychoticism, resilience presented the highest mediating proportion in all three types of SI, whereas for neuroticism, resilience was observed as a comparatively weak mediation. This finding is in accordance with some previously published studies. At first, it has been suggested that extraversion was more closely related to resilience than neuroticism (Jalilianhasanpour et al., 2018). Moreover, Gong et al. (2020) also found a higher mediation proportion of resilience in the association between extroversion and depression than neuroticism-depression association. This finding may indicate that, for children and adolescents, the measuring of personality dimensions can help determine key subpopulations for suicide intervention: for individuals with higher extraversion or psychoticism level, the effect of resilience building measures could be more effective than individuals with higher neuroticism level.

As far as we know, our study is among the first attempts to investigate the mediation of resilience in the association between personality traits and SI among a large population-based sample of Chinese children and adolescents. More importantly, aiming at testing the stability of this mediation, we analyzed three types of SI simultaneously with agreeable results, which further increased the credibility of the conclusion. Our major findings can provide important evidence in devising intervention measures for antagonizing personality-associated suicidal risk in children and adolescents. Nevertheless, our study also has some limitations. First, when analyzing the mediating role of resilience, we used cross-sectional data, and therefore, the findings are only preliminary and should be further corroborated by longitudinal studies (Tate, 2015). However, as personality traits are innate characteristics of individuals, the controversy in the sequence of association should be minimal. Second, we used a self-report questionnaire to collect relevant information from the participants, and therefore, the risk of information bias existed.

## Data Availability Statement

The raw data supporting the conclusions of this article will be made available by the authors, without undue reservation.

## Ethics Statement

The studies involving human participants were reviewed and approved by the Ethics Review Board of Kunming Medical University. Written informed consent to participate in this study was provided by the participants’ legal guardian/next of kin.

## Author Contributions

YX conceived the study. YC, DF, LCh, HR, LCa, SW, JP, HS, and XL collected, verified, and analyzed the data. YC, DF, and LCa drafted the manuscript. All authors provided critical revision of the manuscript for important intellectual content.

## Conflict of Interest

The authors declare that the research was conducted in the absence of any commercial or financial relationships that could be construed as a potential conflict of interest.

## Publisher’s Note

All claims expressed in this article are solely those of the authors and do not necessarily represent those of their affiliated organizations, or those of the publisher, the editors and the reviewers. Any product that may be evaluated in this article, or claim that may be made by its manufacturer, is not guaranteed or endorsed by the publisher.

## Abbreviations

SI: suicide idea
BSSI: Beck Scale for Suicide Ideation
RSCA: Resilience Scale for Chinese Adolescents
EPQ: Eysenck Personality Questionnaires
SH: self-harm
MASHS: The Modified version of Adolescents Self-Harm Scale
PHQ-9: The Patient Health Questionnaire-9
GAD-7: The Generalized Anxiety.
