# Supplementary material for: Mediation of Resilience in the Association Between Personality Traits and Suicidal Ideation Among Chinese Adolescents
Source: Front Psychol. 2022 Jun 3;13:898318. doi: 10.3389/fpsyg.2022.898318 (PMC9204167; doi:10.3389/fpsyg.2022.898318)
Supplement: Supplementary file 1 [file Table_1.docx]

Table S1 Univariate and multivariable Logistic regression models fitting results on associated factors of resilience.

| Covariates | Higher level of resilience | |
| --- | --- | --- |
|  | Univariate  OR (90% CI) | Multivariate  OR (95% CI) |
| Sex (Ref: Boys): Girls | 1.03 (0.88, 1.19) |  |
| Ethnicity (Ref: Han majority) |  |  |
| Yi minority | 0.77 (0.62,0.96) | 0.88 (0.68,1.13) |
| Hui minority | 0.86 (0.64,1.16) | 0.89 (0.58,1.36) |
| Miao minority | 0.66 (0.47,0.92) | 0.87 (0.57,1.31) |
| Other minorities | 0.85 (0.70,1.02) | 0.87 (0.59,1.27) |
| Age: +1 year | 1.01 (0.93, 1.10) |  |
| Grade (Ref: Primary school) |  |  |
| Middle school | 0.77 (0.59, 1.01) |  |
| High school | 1.45 (0.94, 2.23) |  |
| Single child (Ref: Yes): No | 0.72 (0.59, 0.87) | 0.84 (0.67, 1.04) |
| Father’s education level (Ref: Illiteracy) |  |  |
| Primary school | 0.83 (0.66, 1.19) | 0.78 (0.51, 1.17) |
| Middle or high school | 1.28 (0.97, 1.67) | 1.01 (0.71, 1.44) |
| College degree or above | 1.53 (1.02, 2.29) | 0.96 (0.56, 1.65) |
| Father’ age: +1 year | 1.06 (0.99, 1.14) |  |
| Mother’s education level (Ref: Illiteracy) |  |  |
| Primary school | 0.98 (0.85, 1.04) | 0.91 (0.72, 1.14) |
| Middle or high school | 1.45 (1.11, 1.90) | 1.05 (0.74, 1.49) |
| College or above | 1.49 (1.07, 2.06) | 0.97 (0.58, 1.61) |
| Mother’s age: +1 year | 1.01 (0.94, 1.10) |  |
| Marital status of the parents (Ref: In marriage) |  |  |
| Divorced or widowed | 0.84 (0.71, 0.99) | 0.79 (0.52, 1.21) |
| Remarried with someone else | 0.90 (0.72, 1.12) | 1.04 (0.71, 1.52) |
| Depression (Ref: PHQ-9<5): PHQ-9≥5 | 0.31 (0.27, 0.36) | 0.78 (0.38, 0.59) |
| Anxiety (Ref: GAD-7): GAD-7≥5 | 0.23 (0.20, 0.27) | 0.39 (0.26, 0.60) |
| Self-harm (Ref: No): Yes | 0.31 (0.27, 0.36) | 0.51 (0.45, 0.58) |
| Temperament types (Ref: Middle type) |  |  |
| Choleric | 0.50 (0.43, 0.58) | 0.73 (0.62, 0.87) |
| Sanguineous | 4.04 (3.47, 4.70) | 4.10 (3.26, 5.16) |
| Phlegmatic | 0.32 (0.28, 0.37) | 0.31 (0.23, 0.43) |
| Melancholic | 0.08 (0.06, 0.11) | 0.15 (0.10, 0.22) |
